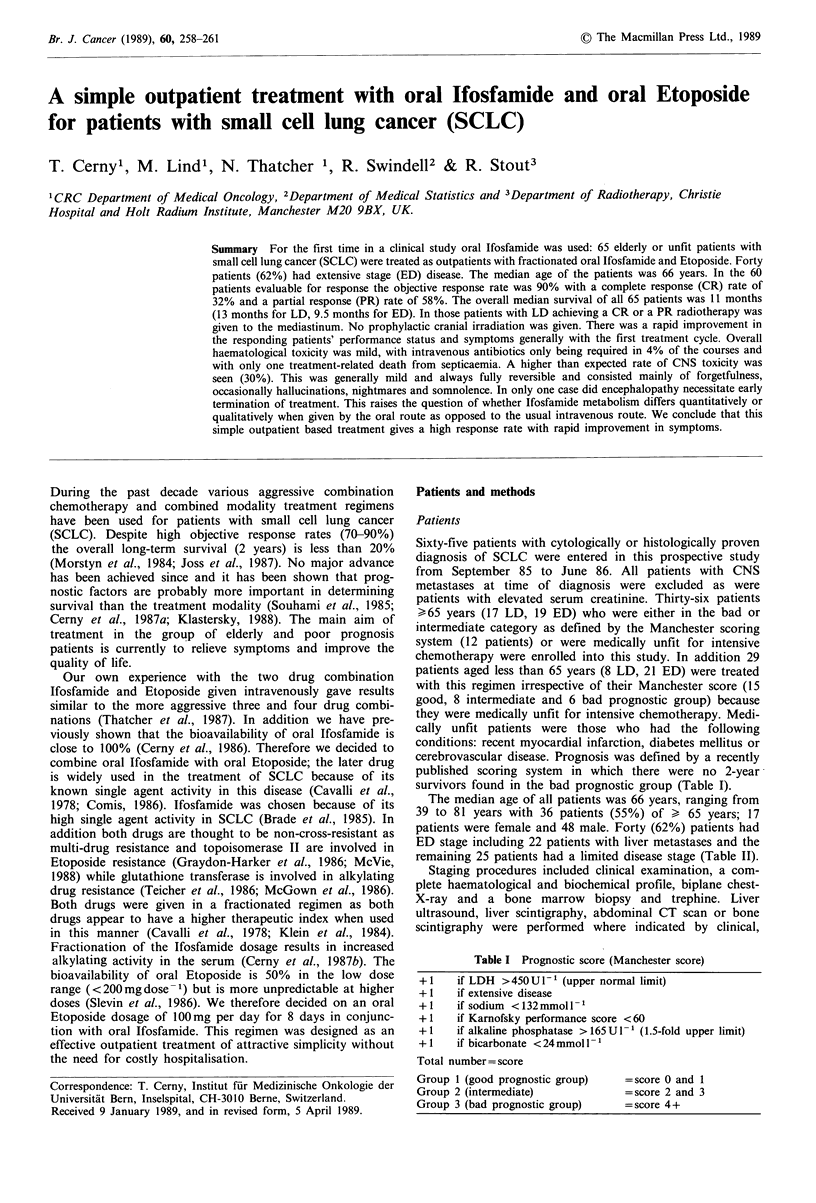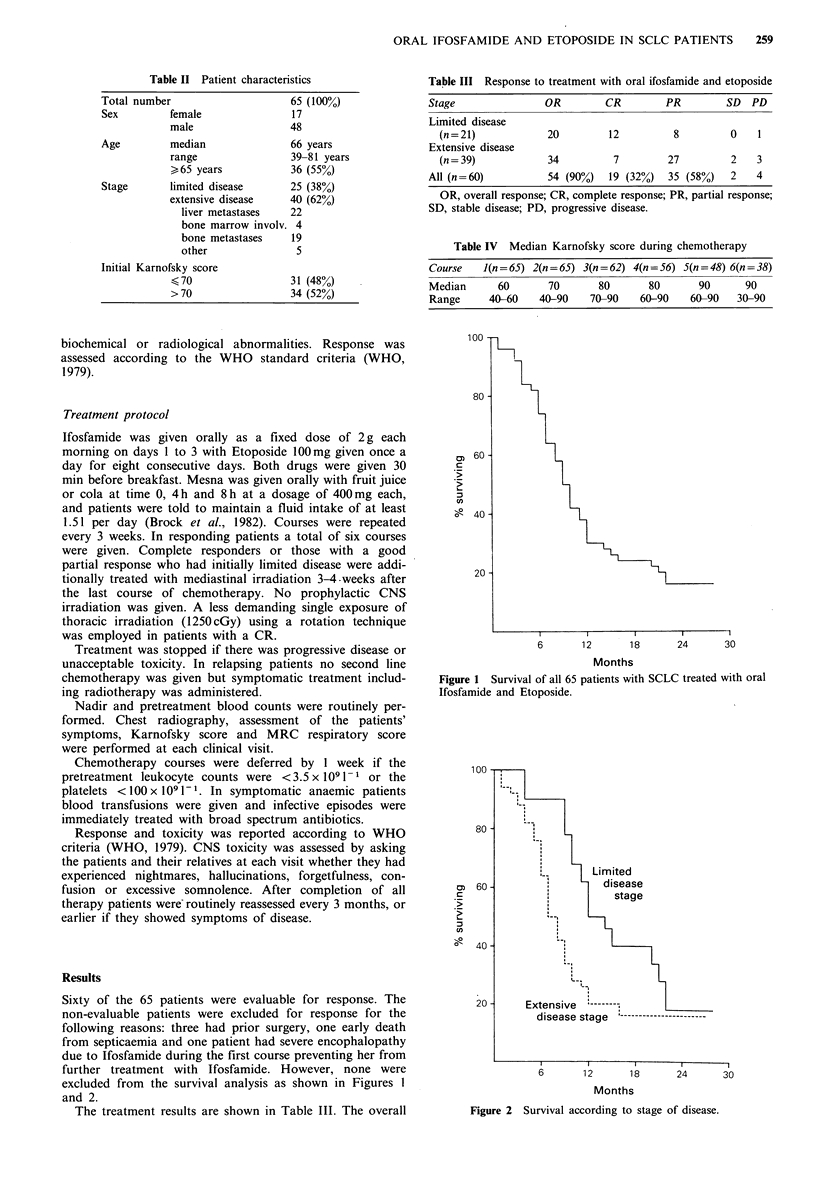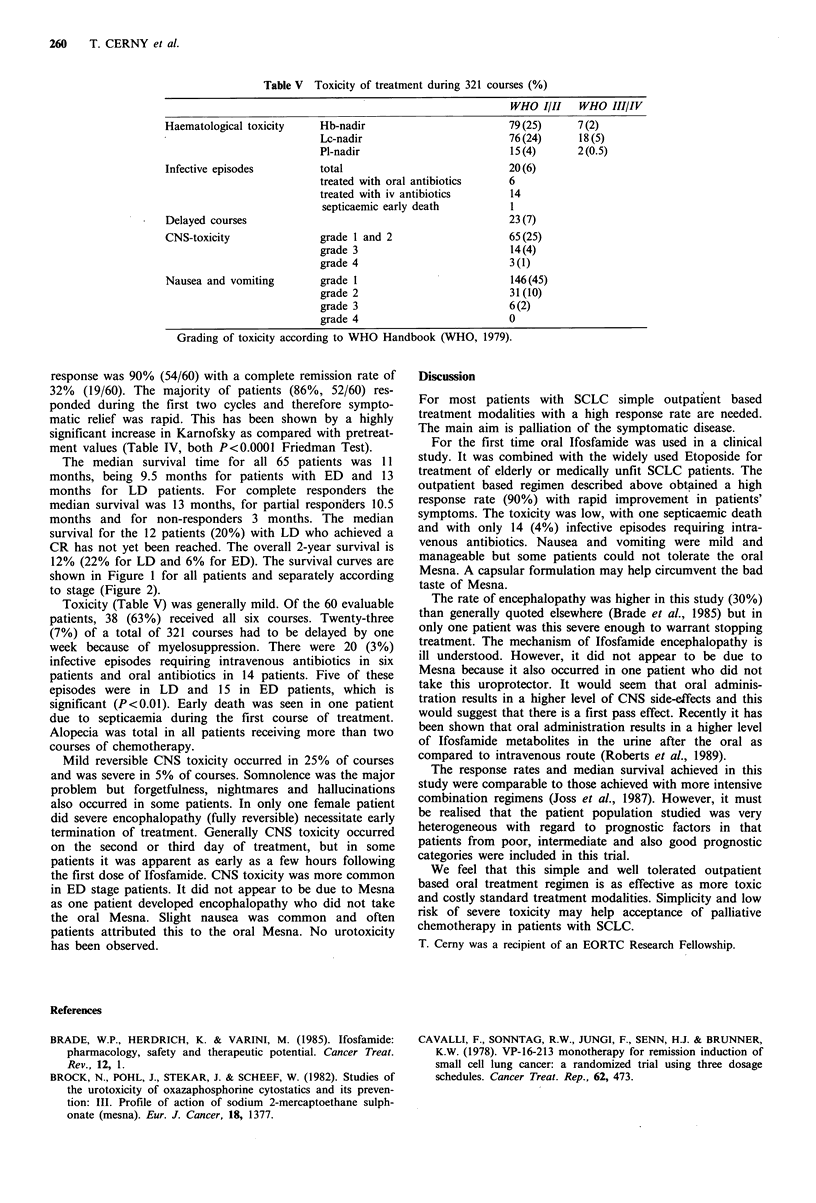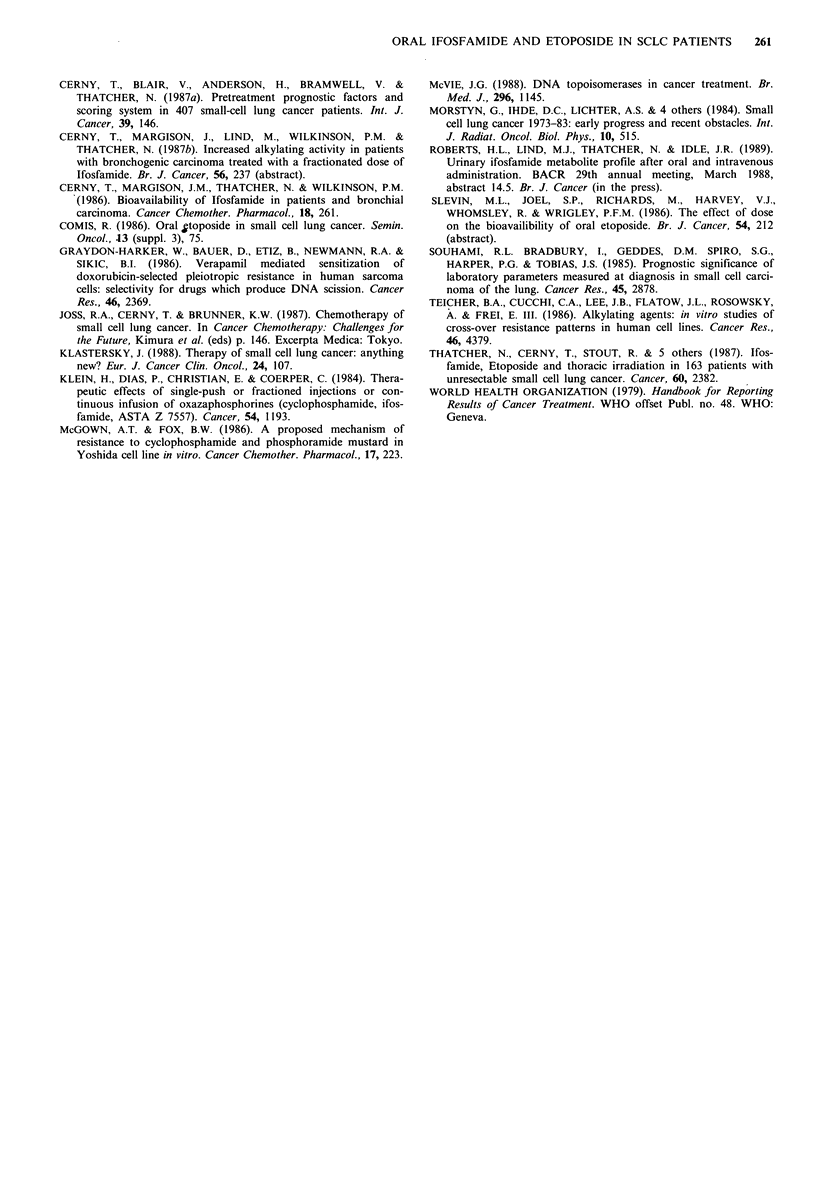# A simple outpatient treatment with oral ifosfamide and oral etoposide for patients with small cell lung cancer (SCLC).

**DOI:** 10.1038/bjc.1989.265

**Published:** 1989-08

**Authors:** T. Cerny, M. Lind, N. Thatcher, R. Swindell, R. Stout

**Affiliations:** CRC Department of Medical Oncology, Christie Hospital and Holt Radium Institute, Manchester, UK.

## Abstract

For the first time in a clinical study oral Ifosfamide was used: 65 elderly or unfit patients with small cell lung cancer (SCLC) were treated as outpatients with fractionated oral Ifosfamide and Etoposide. Forty patients (62%) had extensive stage (ED) disease. The median age of the patients was 66 years. In the 60 patients evaluable for response the objective response rate was 90% with a complete response (CR) rate of 32% and a partial response (PR) rate of 58%. The overall median survival of all 65 patients was 11 months (13 months for LD, 9.5 months for ED). In those patients with LD achieving a CR or a PR radiotherapy was given to the mediastinum. No prophylactic cranial irradiation was given. There was a rapid improvement in the responding patients' performance status and symptoms generally with the first treatment cycle. Overall haematological toxicity was mild, with intravenous antibiotics only being required in 4% of the courses and with only one treatment-related death from septicaemia. A higher than expected rate of CNS toxicity was seen (30%). This was generally mild and always fully reversible and consisted mainly of forgetfulness, occasionally hallucinations, nightmares and somnolence. In only one case did encephalopathy necessitate early termination of treatment. This raises the question of whether Ifosfamide metabolism differs quantitatively or qualitatively when given by the oral route as opposed to the usual intravenous route. We conclude that this simple outpatient based treatment gives a high response rate with rapid improvement in symptoms.


Br. J. Cancer (1989), 60, 258-261 © The Macmillan Press Ltd., 1989

### A simple outpatient treatment with oral Ifosfamide and oral Etoposide for patients with small cell lung cancer (SCLC)

T. Cerny[1], M. Lind[1], N. Thatcher [1], R. Swindell[2] & R. Stout[3]

[1]*CRC Department of Medical Oncology,* [2]*Department of Medical Statistics and* [3]*Department of Radiotherapy, Christie Hospital and Holt Radium Institute, Manchester M20 9BX, UK.*

**Summary** For the first time in a clinical study oral Ifosfamide was used: 65 elderly or unfit patients with small cell lung cancer (SCLC) were treated as outpatients with fractionated oral Ifosfamide and Etoposide. Forty patients (62%) had extensive stage (ED) disease. The median age of the patients was 66 years. In the 60 patients evaluable for response the objective response rate was 90% with a complete response (CR) rate of 32% and a partial response (PR) rate of 58%. The overall median survival of all 65 patients was 11 months (13 months for LD, 9.5 months for ED). In those patients with LD achieving a CR or a PR radiotherapy was given to the mediastinum. No prophylactic cranial irradiation was given. There was a rapid improvement in the responding patients' performance status and symptoms generally with the first treatment cycle. Overall haematological toxicity was mild, with intravenous antibiotics only being required in 4% of the courses and with only one treatment-related death from septicaemia. A higher than expected rate of CNS toxicity was seen (30%). This was generally mild and always fully reversible and consisted mainly of forgetfulness, occasionally hallucinations, nightmares and somnolence. In only one case did encephalopathy necessitate early termination of treatment. This raises the question of whether Ifosfamide metabolism differs quantitatively or qualitatively when given by the oral route as opposed to the usual intravenous route. We conclude that this simple outpatient based treatment gives a high response rate with rapid improvement in symptoms.

During the past decade various aggressive combination chemotherapy and combined modality treatment regimens have been used for patients with small cell lung cancer (SCLC). Despite high objective response rates (70–90%) the overall long-term survival (2 years) is less than 20% (Morstyn *et al.*, 1984; Joss *et al.*, 1987). No major advance has been achieved since and it has been shown that prognostic factors are probably more important in determining survival than the treatment modality (Souhami *et al.*, 1985; Cerny *et al.*, 1987*a*; Klastersky, 1988). The main aim of treatment in the group of elderly and poor prognosis patients is currently to relieve symptoms and improve the quality of life.

Our own experience with the two drug combination Ifosfamide and Etoposide given intravenously gave results similar to the more aggressive three and four drug combinations (Thatcher *et al.*, 1987). In addition we have previously shown that the bioavailability of oral Ifosfamide is close to 100% (Cerny *et al.*, 1986). Therefore we decided to combine oral Ifosfamide with oral Etoposide; the later drug is widely used in the treatment of SCLC because of its known single agent activity in this disease (Cavalli *et al.*, 1978; Comis, 1986). Ifosfamide was chosen because of its high single agent activity in SCLC (Brade *et al.*, 1985). In addition both drugs are thought to be non-cross-resistant as multi-drug resistance and topoisomerase II are involved in Etoposide resistance (Graydon-Harker *et al.*, 1986; McVie, 1988) while glutathione transferase is involved in alkylating drug resistance (Teicher *et al.*, 1986; McGown *et al.*, 1986). Both drugs were given in a fractionated regimen as both drugs appear to have a higher therapeutic index when used in this manner (Cavalli *et al.*, 1978; Klein *et al.*, 1984). Fractionation of the Ifosfamide dosage results in increased alkylating activity in the serum (Cerny *et al.*, 1987*b*). The bioavailability of oral Etoposide is 50% in the low dose range ($<200\,\mathrm{mg\,dose^{-1}}$) but is more unpredictable at higher doses (Slevin *et al.*, 1986). We therefore decided on an oral Etoposide dosage of 100 mg per day for 8 days in conjunction with oral Ifosfamide. This regimen was designed as an effective outpatient treatment of attractive simplicity without the need for costly hospitalisation.

Correspondence: T. Cerny, Institut für Medizinische Onkologie der Universität Bern, Inselspital, CH-3010 Berne, Switzerland.
Received 9 January 1989, and in revised form, 5 April 1989.

### Patients and methods

### *Patients*

Sixty-five patients with cytologically or histologically proven diagnosis of SCLC were entered in this prospective study from September 85 to June 86. All patients with CNS metastases at time of diagnosis were excluded as were patients with elevated serum creatinine. Thirty-six patients ⩾65 years (17 LD, 19 ED) who were either in the bad or intermediate category as defined by the Manchester scoring system (12 patients) or were medically unfit for intensive chemotherapy were enrolled into this study. In addition 29 patients aged less than 65 years (8 LD, 21 ED) were treated with this regimen irrespective of their Manchester score (15 good, 8 intermediate and 6 bad prognostic group) because they were medically unfit for intensive chemotherapy. Medically unfit patients were those who had the following conditions: recent myocardial infarction, diabetes mellitus or cerebrovascular disease. Prognosis was defined by a recently published scoring system in which there were no 2-year survivors found in the bad prognostic group (Table I).

The median age of all patients was 66 years, ranging from 39 to 81 years with 36 patients (55%) of ⩾ 65 years; 17 patients were female and 48 male. Forty (62%) patients had ED stage including 22 patients with liver metastases and the remaining 25 patients had a limited disease stage (Table II).

Staging procedures included clinical examination, a complete haematological and biochemical profile, biplane chest-X-ray and a bone marrow biopsy and trephine. Liver ultrasound, liver scintigraphy, abdominal CT scan or bone scintigraphy were performed where indicated by clinical,

**Table I** Prognostic score (Manchester score)

| | |
|---|---|
| +1 | if LDH $>450\,\mathrm{U\,l^{-1}}$ (upper normal limit) |
| +1 | if extensive disease |
| +1 | if sodium $<132\,\mathrm{mmol\,l^{-1}}$ |
| +1 | if Karnofsky performance score <60 |
| +1 | if alkaline phosphatase $>165\,\mathrm{U\,l^{-1}}$ (1.5-fold upper limit) |
| +1 | if bicarbonate $<24\,\mathrm{mmol\,l^{-1}}$ |
| Total number = score | |
| Group 1 (good prognostic group) | = score 0 and 1 |
| Group 2 (intermediate) | = score 2 and 3 |
| Group 3 (bad prognostic group) | = score 4+ |

**Table II** Patient characteristics

| | | |
|---|---|---|
| Total number | | 65 (100%) |
| Sex | female | 17 |
| | male | 48 |
| Age | median | 66 years |
| | range | 39–81 years |
| | ≥65 years | 36 (55%) |
| Stage | limited disease | 25 (38%) |
| | extensive disease | 40 (62%) |
| | liver metastases | 22 |
| | bone marrow involv. | 4 |
| | bone metastases | 19 |
| | other | 5 |
| Initial Karnofsky score | | |
| | ≤70 | 31 (48%) |
| | >70 | 34 (52%) |

biochemical or radiological abnormalities. Response was assessed according to the WHO standard criteria (WHO, 1979).

*Treatment protocol*

Ifosfamide was given orally as a fixed dose of 2 g each morning on days 1 to 3 with Etoposide 100 mg given once a day for eight consecutive days. Both drugs were given 30 min before breakfast. Mesna was given orally with fruit juice or cola at time 0, 4 h and 8 h at a dosage of 400 mg each, and patients were told to maintain a fluid intake of at least 1.5 l per day (Brock *et al.*, 1982). Courses were repeated every 3 weeks. In responding patients a total of six courses were given. Complete responders or those with a good partial response who had initially limited disease were additionally treated with mediastinal irradiation 3–4 weeks after the last course of chemotherapy. No prophylactic CNS irradiation was given. A less demanding single exposure of thoracic irradiation (1250 cGy) using a rotation technique was employed in patients with a CR.

Treatment was stopped if there was progressive disease or unacceptable toxicity. In relapsing patients no second line chemotherapy was given but symptomatic treatment including radiotherapy was administered.

Nadir and pretreatment blood counts were routinely performed. Chest radiography, assessment of the patients' symptoms, Karnofsky score and MRC respiratory score were performed at each clinical visit.

Chemotherapy courses were deferred by 1 week if the pretreatment leukocyte counts were $<3.5 \times 10^9 l^{-1}$ or the platelets $<100 \times 10^9 l^{-1}$. In symptomatic anaemic patients blood transfusions were given and infective episodes were immediately treated with broad spectrum antibiotics.

Response and toxicity was reported according to WHO criteria (WHO, 1979). CNS toxicity was assessed by asking the patients and their relatives at each visit whether they had experienced nightmares, hallucinations, forgetfulness, confusion or excessive somnolence. After completion of all therapy patients were routinely reassessed every 3 months, or earlier if they showed symptoms of disease.

### Results

Sixty of the 65 patients were evaluable for response. The non-evaluable patients were excluded for response for the following reasons: three had prior surgery, one early death from septicaemia and one patient had severe encophalopathy due to Ifosfamide during the first course preventing her from further treatment with Ifosfamide. However, none were excluded from the survival analysis as shown in Figures 1 and 2.

The treatment results are shown in Table III. The overall

**Table III** Response to treatment with oral ifosfamide and etoposide

| *Stage* | *OR* | *CR* | *PR* | *SD* | *PD* |
|---|---|---|---|---|---|
| Limited disease ($n=21$) | 20 | 12 | 8 | 0 | 1 |
| Extensive disease ($n=39$) | 34 | 7 | 27 | 2 | 3 |
| All ($n=60$) | 54 (90%) | 19 (32%) | 35 (58%) | 2 | 4 |

OR, overall response; CR, complete response; PR, partial response; SD, stable disease; PD, progressive disease.

**Table IV** Median Karnofsky score during chemotherapy

| *Course* | *1(n=65)* | *2(n=65)* | *3(n=62)* | *4(n=56)* | *5(n=48)* | *6(n=38)* |
|---|---|---|---|---|---|---|
| Median | 60 | 70 | 80 | 80 | 90 | 90 |
| Range | 40–60 | 40–90 | 70–90 | 60–90 | 60–90 | 30–90 |

**Figure 1** Survival of all 65 patients with SCLC treated with oral Ifosfamide and Etoposide.

**Figure 2** Survival according to stage of disease.

**Table V** Toxicity of treatment during 321 courses (%)

| | | *WHO I/II* | *WHO III/IV* |
|---|---|---|---|
| Haematological toxicity | Hb-nadir | 79 (25) | 7 (2) |
| | Lc-nadir | 76 (24) | 18 (5) |
| | Pl-nadir | 15 (4) | 2 (0.5) |
| Infective episodes | total | 20 (6) | |
| | treated with oral antibiotics | 6 | |
| | treated with iv antibiotics | 14 | |
| | septicaemic early death | 1 | |
| Delayed courses | | 23 (7) | |
| CNS-toxicity | grade 1 and 2 | 65 (25) | |
| | grade 3 | 14 (4) | |
| | grade 4 | 3 (1) | |
| Nausea and vomiting | grade 1 | 146 (45) | |
| | grade 2 | 31 (10) | |
| | grade 3 | 6 (2) | |
| | grade 4 | 0 | |

Grading of toxicity according to WHO Handbook (WHO, 1979).

response was 90% (54/60) with a complete remission rate of 32% (19/60). The majority of patients (86%, 52/60) responded during the first two cycles and therefore symptomatic relief was rapid. This has been shown by a highly significant increase in Karnofsky as compared with pretreatment values (Table IV, both $P<0.0001$ Friedman Test).

The median survival time for all 65 patients was 11 months, being 9.5 months for patients with ED and 13 months for LD patients. For complete responders the median survival was 13 months, for partial responders 10.5 months and for non-responders 3 months. The median survival for the 12 patients (20%) with LD who achieved a CR has not yet been reached. The overall 2-year survival is 12% (22% for LD and 6% for ED). The survival curves are shown in Figure 1 for all patients and separately according to stage (Figure 2).

Toxicity (Table V) was generally mild. Of the 60 evaluable patients, 38 (63%) received all six courses. Twenty-three (7%) of a total of 321 courses had to be delayed by one week because of myelosuppression. There were 20 (3%) infective episodes requiring intravenous antibiotics in six patients and oral antibiotics in 14 patients. Five of these episodes were in LD and 15 in ED patients, which is significant ($P<0.01$). Early death was seen in one patient due to septicaemia during the first course of treatment. Alopecia was total in all patients receiving more than two courses of chemotherapy.

Mild reversible CNS toxicity occurred in 25% of courses and was severe in 5% of courses. Somnolence was the major problem but forgetfulness, nightmares and hallucinations also occurred in some patients. In only one female patient did severe encophalopathy (fully reversible) necessitate early termination of treatment. Generally CNS toxicity occurred on the second or third day of treatment, but in some patients it was apparent as early as a few hours following the first dose of Ifosfamide. CNS toxicity was more common in ED stage patients. It did not appear to be due to Mesna as one patient developed encophalopathy who did not take the oral Mesna. Slight nausea was common and often patients attributed this to the oral Mesna. No urotoxicity has been observed.

### Discussion

For most patients with SCLC simple outpatient based treatment modalities with a high response rate are needed. The main aim is palliation of the symptomatic disease.

For the first time oral Ifosfamide was used in a clinical study. It was combined with the widely used Etoposide for treatment of elderly or medically unfit SCLC patients. The outpatient based regimen described above obtained a high response rate (90%) with rapid improvement in patients' symptoms. The toxicity was low, with one septicaemic death and with only 14 (4%) infective episodes requiring intravenous antibiotics. Nausea and vomiting were mild and manageable but some patients could not tolerate the oral Mesna. A capsular formulation may help circumvent the bad taste of Mesna.

The rate of encephalopathy was higher in this study (30%) than generally quoted elsewhere (Brade *et al.*, 1985) but in only one patient was this severe enough to warrant stopping treatment. The mechanism of Ifosfamide encephalopathy is ill understood. However, it did not appear to be due to Mesna because it also occurred in one patient who did not take this uroprotector. It would seem that oral administration results in a higher level of CNS side-effects and this would suggest that there is a first pass effect. Recently it has been shown that oral administration results in a higher level of Ifosfamide metabolites in the urine after the oral as compared to intravenous route (Roberts *et al.*, 1989).

The response rates and median survival achieved in this study were comparable to those achieved with more intensive combination regimens (Joss *et al.*, 1987). However, it must be realised that the patient population studied was very heterogeneous with regard to prognostic factors in that patients from poor, intermediate and also good prognostic categories were included in this trial.

We feel that this simple and well tolerated outpatient based oral treatment regimen is as effective as more toxic and costly standard treatment modalities. Simplicity and low risk of severe toxicity may help acceptance of palliative chemotherapy in patients with SCLC.

T. Cerny was a recipient of an EORTC Research Fellowship.

### References

BRADE, W.P., HERDRICH, K. & VARINI, M. (1985). Ifosfamide: pharmacology, safety and therapeutic potential. *Cancer Treat. Rev.*, **12**, 1.

BROCK, N., POHL, J., STEKAR, J. & SCHEEF, W. (1982). Studies of the urotoxicity of oxazaphosphorine cytostatics and its prevention: III. Profile of action of sodium 2-mercaptoethane sulphonate (mesna). *Eur. J. Cancer*, **18**, 1377.

CAVALLI, F., SONNTAG, R.W., JUNGI, F., SENN, H.J. & BRUNNER, K.W. (1978). VP-16-213 monotherapy for remission induction of small cell lung cancer: a randomized trial using three dosage schedules. *Cancer Treat. Rep.*, **62**, 473.

CERNY, T., BLAIR, V., ANDERSON, H., BRAMWELL, V. & THATCHER, N. (1987*a*). Pretreatment prognostic factors and scoring system in 407 small-cell lung cancer patients. *Int. J. Cancer*, **39**, 146.

CERNY, T., MARGISON, J., LIND, M., WILKINSON, P.M. & THATCHER, N. (1987*b*). Increased alkylating activity in patients with bronchogenic carcinoma treated with a fractionated dose of Ifosfamide. *Br. J. Cancer*, **56**, 237 (abstract).

CERNY, T., MARGISON, J.M., THATCHER, N. & WILKINSON, P.M. (1986). Bioavailability of Ifosfamide in patients and bronchial carcinoma. *Cancer Chemother. Pharmacol.*, **18**, 261.

COMIS, R. (1986). Oral etoposide in small cell lung cancer. *Semin. Oncol.*, **13** (suppl. 3), 75.

GRAYDON-HARKER, W., BAUER, D., ETIZ, B., NEWMANN, R.A. & SIKIC, B.I. (1986). Verapamil mediated sensitization of doxorubicin-selected pleiotropic resistance in human sarcoma cells: selectivity for drugs which produce DNA scission. *Cancer Res.*, **46**, 2369.

JOSS, R.A., CERNY, T. & BRUNNER, K.W. (1987). Chemotherapy of small cell lung cancer. In *Cancer Chemotherapy: Challenges for the Future*, Kimura *et al.* (eds) p. 146. Excerpta Medica: Tokyo.

KLASTERSKY, J. (1988). Therapy of small cell lung cancer: anything new? *Eur. J. Cancer Clin. Oncol.*, **24**, 107.

KLEIN, H., DIAS, P., CHRISTIAN, E. & COERPER, C. (1984). Therapeutic effects of single-push or fractioned injections or continuous infusion of oxazaphosphorines (cyclophosphamide, ifosfamide, ASTA Z 7557). *Cancer*, **54**, 1193.

McGOWN, A.T. & FOX, B.W. (1986). A proposed mechanism of resistance to cyclophosphamide and phosphoramide mustard in Yoshida cell line *in vitro*. *Cancer Chemother. Pharmacol.*, **17**, 223.

McVIE, J.G. (1988). DNA topoisomerases in cancer treatment. *Br. Med. J.*, **296**, 1145.

MORSTYN, G., IHDE, D.C., LICHTER, A.S. & 4 others (1984). Small cell lung cancer 1973–83: early progress and recent obstacles. *Int. J. Radiat. Oncol. Biol. Phys.*, **10**, 515.

ROBERTS, H.L., LIND, M.J., THATCHER, N. & IDLE, J.R. (1989). Urinary ifosfamide metabolite profile after oral and intravenous administration. BACR 29th annual meeting, March 1988, abstract 14.5. *Br. J. Cancer* (in the press).

SLEVIN, M.L., JOEL, S.P., RICHARDS, M., HARVEY, V.J., WHOMSLEY, R. & WRIGLEY, P.F.M. (1986). The effect of dose on the bioavailibility of oral etoposide. *Br. J. Cancer*, **54**, 212 (abstract).

SOUHAMI, R.L. BRADBURY, I., GEDDES, D.M. SPIRO, S.G., HARPER, P.G. & TOBIAS, J.S. (1985). Prognostic significance of laboratory parameters measured at diagnosis in small cell carcinoma of the lung. *Cancer Res.*, **45**, 2878.

TEICHER, B.A., CUCCHI, C.A., LEE, J.B., FLATOW, J.L., ROSOWSKY, A. & FREI, E. III. (1986). Alkylating agents: *in vitro* studies of cross-over resistance patterns in human cell lines. *Cancer Res.*, **46**, 4379.

THATCHER, N., CERNY, T., STOUT, R. & 5 others (1987). Ifosfamide, Etoposide and thoracic irradiation in 163 patients with unresectable small cell lung cancer. *Cancer*, **60**, 2382.

WORLD HEALTH ORGANIZATION (1979). *Handbook for Reporting Results of Cancer Treatment*. WHO offset Publ. no. 48. WHO: Geneva.